# A New Model for Youth-Driven Community Change: Exploratory Testing of Artificial Intelligence–Supported Citizen Science

**DOI:** 10.2196/79464

**Published:** 2026-04-29

**Authors:** Eduardo De la Vega-Taboada, Sofia A Portillo, Lina Maria Gomez-Garcia, Ann Banchoff, Victoria Maria Bermudez, Diana M Chavez, Tate Isabella Sgaraglino, Eugenia Flores Millender, Olga L Sarmiento, Abby C King

**Affiliations:** 1 Department of Epidemiology & Population Health School of Medicine Stanford University Stanford, CA United States; 2 College of Nursing Center of Population Science for Health Empowerment Florida State University Tallahassee, FL United States; 3 School of Medicine Universidad de Los Andes Bogota Colombia; 4 Department of Medicine (Stanford Prevention Research Center) School of Medicine Stanford University Stanford, CA United States

**Keywords:** youth-led citizen sciences, large language models, GPT, health-environment interaction, stakeholder engagement, action research

## Abstract

**Background:**

Generative artificial intelligence (AI) systems are increasingly used in health and community settings, yet empirical evidence on how they function within participatory, youth-led action frameworks remains limited. Large language models can provide structured feedback to support planning and critical reflection, and AI-based image transformation can generate realistic visual prototypes to enhance shared understanding. However, risks include output variability, feasibility gaps when AI-generated recommendations or visualizations imply solutions that are not operationally workable, and the potential to displace adolescent voice and agency if AI outputs are treated as authoritative rather than as inputs for collective deliberation.

**Objective:**

This study examines how 2 generative AI tools—structured feedback using a GPT model and AI-based image transformation—functioned as deliberative and visualization supports within a youth-led citizen science intervention addressing environmental health concerns in El Pozón, Cartagena, Colombia.

**Methods:**

This exploratory action research study included a preparation phase and an implementation phase. During preparation, researchers iteratively tested SecureGPT (a privacy-enhanced version of ChatGPT 4.0) prompt configurations and compared DALL-E with Adobe Photoshop AI for place-based image modification, selecting a fixed prompt format requesting 3 strengths, 3 weaknesses, and 5 reflective questions (3-3-5). During implementation, 12 adolescent citizen scientists completed the *Our Voice* process. AI use was facilitator-mediated: prompts were co-developed through youth consensus, a facilitator entered prompts and operated tools while youth observed, and outputs were reviewed with the group in real time before use. Data sources included structured field notes, analytic memos, archived prompts and outputs, and session recordings. Analysis was descriptive and process-oriented, examining how AI shaped deliberation, solution refinement, and stakeholder engagement.

**Results:**

Structured GPT prompts supported deeper critical analysis and iterative refinement toward more feasible interventions. Model outputs varied in usefulness; role-based prompting often produced redundant responses, and early outputs were occasionally overly generic, requiring facilitator guidance and prompt refinement. The structured 3-3-5 format improved specificity and reduced wordiness. DALL-E did not generate sufficiently realistic place-based modifications, whereas Adobe Photoshop AI, used with iterative prompting and area-selection tools, produced visually plausible prototypes that supported group discussion and stakeholder communication. Highly realistic visualizations also introduced a feasibility gap when depicted infrastructure exceeded operational constraints, requiring explicit framing of images as aspirational prototypes rather than technical designs.

**Conclusions:**

In this facilitated participatory context, generative AI tools served as structured deliberative and visualization supports rather than autonomous decision makers. For participatory action and citizen science researchers, these findings suggest a practical workflow in which structured prompting, real-time group review, and domain-informed oversight can help participants refine feasible solutions, strengthen communication with stakeholders, and document iterative decision-making while managing variability, accuracy, privacy, and feasibility alignment.

## Introduction

### Background

The intricate relationship between health and the environment is well established, particularly for global youth. Adolescents experiencing environmental challenges in their communities, such as poor waste management or stagnant water in the streets, are more likely to develop diseases such as dengue fever, Zika, parasitic diarrhea, and acute dermatitis, as well as mental health issues such as anxiety and depression [1,2]. For example, research in Indonesia showed that children living in households where trash was not routinely collected and removed were significantly more likely to be dengue seropositive [2]. These hazards often reflect persistent infrastructure deficits such as poor drainage, insufficient waste services, and limited maintenance of public spaces, which can accumulate over time and reinforce long-term health inequities. Despite widespread recognition of these risks, many communities lack the resources, authority, or partnerships needed to translate knowledge into sustained environmental improvements [3,4]. This gap is especially pronounced in low- and middle-income settings, where government capacity to implement meaningful change is frequently constrained [5].

Youth-led citizen science strategies aimed at enhancing local environments have demonstrated significant benefits, including improved health outcomes and increased feelings of empowerment among adolescents [6,7]. These strategies enable adolescents to become investigators of their environment and promoters of positive change. However, successful implementation of these interventions is challenging, as it requires meaningful engagement from adolescents, strong stakeholder commitment, and well-planned, feasible, and efficient solutions [2,7].

To increase appeal and impact, efforts to engage youth in community solution-building, using approaches such as the *Our Voice* method [8], can leverage the digital technologies that young people around the world engage with fluently on a daily basis [9]. Such technologies are evolving rapidly, characterized by the widespread adoption of artificial intelligence (AI), which has demonstrated an increasing capacity to process complex data, transform images, and generate insights to solve problems. AI can model environmental changes with a level of detail and accuracy previously unattainable, enabling more informed decision-making that could lead to more effective interventions, such as optimizing renewable energy resources, improving waste management, and predicting natural disasters [10-12]. Additionally, AI has the potential to provide tools that enhance action-oriented citizen science and transform adolescents’ environments.

This AI application is ready for broad exploration, and several tools offer opportunities for initial testing of potential impacts on youth engagement in community health improvement. For example, GPT language models can provide constructive feedback related to solution-building. Furthermore, photo transformation tools can improve community members’ ability to visualize potential solutions and enrich their subsequent engagement with stakeholders. However, these capabilities come with considerable risks, particularly the potential to amplify existing inequalities if AI research and development are concentrated within already privileged populations or contexts [13]. Recent scholarship further cautions that generative AI systems may produce plausible but inaccurate outputs, reflect embedded biases in training data, and require structured human oversight to ensure ethical and contextually appropriate deployment in mental health–related settings [14]. If not applied judiciously, AI use might also risk replacing adolescents’ voices rather than amplifying them [15].

In this study, we adapted and evaluated the use of 2 specific AI tools to support community-driven action research (1) providing feedback through GPT, and (2) image transformation. We then applied these tools to a citizen science project that engaged adolescent residents of the vulnerable neighborhood of El Pozón in Cartagena, Colombia.

### Providing Feedback Through GPTs

OpenAI ChatGPT and similar AI systems can offer critical feedback on problem-solving strategies, especially in community-based initiatives. For instance, adolescents proposing the draining of stagnant water to improve public health in their communities can amplify their impact by leveraging AI to refine their ideas and provide constructive feedback. ChatGPT can assist in identifying potential challenges to proposed strategies, suggesting alternative approaches, and offering evidence-based recommendations to enhance the effectiveness of their solutions. As noted earlier, by engaging with AI, young people can potentially deepen their understanding of public health issues, gain insights into the environmental and social implications of their actions, and develop more comprehensive and sustainable interventions. This collaborative process between AI and human creativity may support communities in addressing local problems more effectively and innovatively [15,16].

### Image Transformation Through AI

AI technologies like Adobe Photoshop AI can reshape our perceptions of the future by transforming visual representations of the world. This technology empowers designers, architects, and urban planners to create compelling visions of sustainable cities, clean energy landscapes, and resilient communities that inspire action and drive societal change. In the context of community-driven change, these tools enable us to reimagine the “undesirable present” and project a “desirable future” with clarity. Such AI-generated imagery may arguably galvanize public support for environmental and social initiatives, making the abstract tangible and the future visible [17].

### Context of the Study: The *Our Voice* Initiative in El Pozón

El Pozón, in Cartagena, Colombia, has witnessed rapid urbanization and glaring social disparities over the last 2 decades. Community members in this area, established as an informal settlement in 1969, still grapple with a lack of essential services, multidimensional poverty, and unfavorable living conditions [18]. In this intricate and challenging context, Bradbury and colleagues [19] made a compelling case for the need to incorporate diverse sources of knowledge to advance positive community-level change. They emphasized that involving individuals intimately connected to the issues is imperative to mitigate the pervasive consequences of poverty and unhealthy environments. In this regard, the constructive collaboration between action research and citizen science emerges as a potent catalyst, harnessing the abundance of local human knowledge and understanding to effectively and sustainably address pressing community problems.

### The *Our Voice* Initiative

The Stanford University *Our Voice* citizen science method is a form of participatory action research that engages community residents as scientists through a multidimensional mixed methods approach [7]. In a 4-step process, participants become citizen scientists through a user-friendly, multilingual mobile app called the Discovery Tool.

Using the Discovery Tool app, citizen scientists take georeferenced photographs and record narratives, either through audio recordings or text notes, describing the elements in their environment that either diminish or enhance their health and well-being. To conclude this Discover step, the citizen scientists upload their anonymized Discovery Tool data to a secure server. Then, during the Discuss step, the citizen scientists participate in facilitated meetings to discuss, analyze, and prioritize their group’s collective findings and then brainstorm potential changes and action steps.

The next step involves 1 or more facilitated meetings in which stakeholders are invited to the citizen scientists’ presentation of their findings and recommendations (advocacy step). Ideally, the citizen scientists then work collaboratively with other community stakeholders to co-organize and implement realistic next steps to address the pressing problems they identified during the *Our Voice* process (change step). While the *Our Voice* platform has been shown to be accessible and effective, to date, the integration of AI to enhance the community-driven change process has not been fully tested [15,20].

### Study Design

This was an action research study aimed at investigating AI’s capacities to further enrich the *Our Voice* process by amplifying and activating the collective voices of vulnerable adolescents (also known as citizen scientists) as they endeavored to improve their community in El Pozón, Colombia.

The study consisted of several phases. First, during a preparation phase, we (the researchers) evaluated the initial applicability of 2 AI tools: image transformation and feedback generation using SecureGPT (a privacy-enhanced version of ChatGPT 4.0; OpenAI). During this phase, we conducted several small experiments, including transforming images with various prompts and refining solutions with SecureGPT. The results of these experiments allowed us to reflect on the effectiveness of the AI tools and determine the best ways to adapt them for real-world applications.

**Figure 1:**
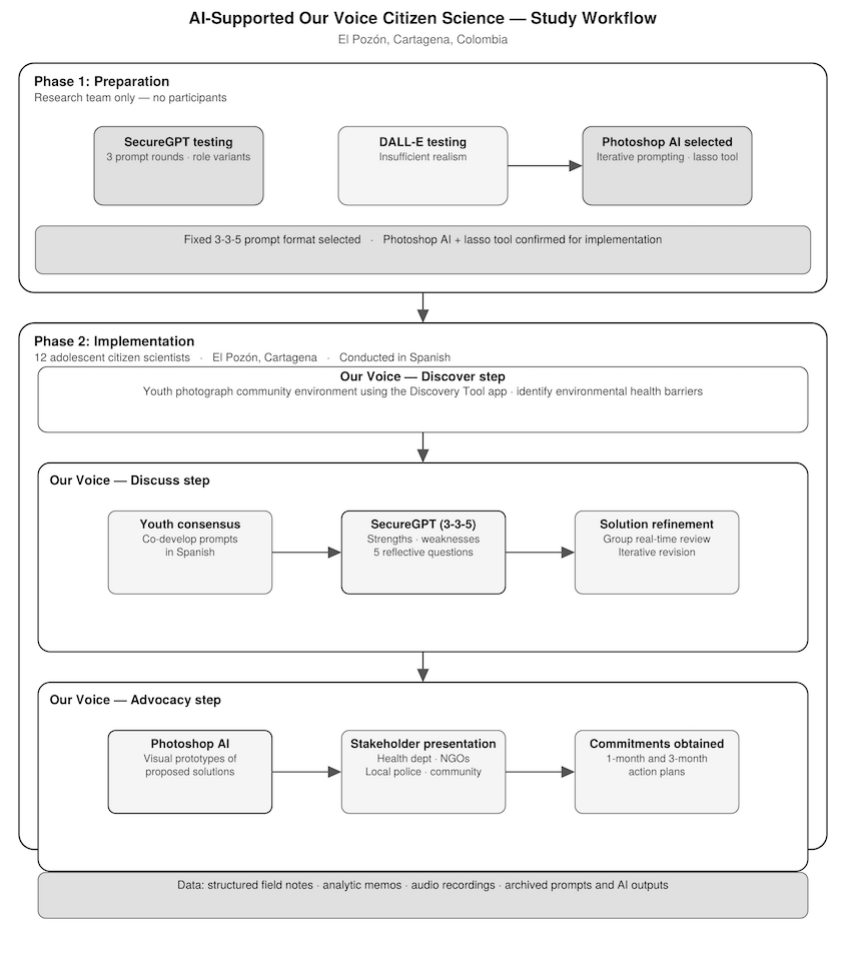
Study flow of the artificial intelligence (AI)–enhanced *Our Voice* process. NGO: nongovernmental organization.

Second, we applied insights from the preparation phase to tailor AI tools for the *Our Voice* project in El Pozón. We used these adapted tools during the *Our Voice* discuss and advocacy steps of the El Pozón project and evaluated their effectiveness in enhancing the *Our Voice* process. Figure 1 provides a schematic overview of the full study workflow.

## Methods

### Study Design

This study consisted of 2 phases. In the first phase, the researchers tested different AI tools to prepare for their use in the second phase. The second phase involved implementing the AI tools in a citizen science project conducted on the Caribbean coast of Colombia.

### Testing of the AI Tools During the Preparation Phase

During the study preparation phase, the researchers tested the integration of AI-supported interactive conversations using Stanford Medicine SecureGPT (beta), a privacy-enhanced version of ChatGPT 4.0. During this phase, which did not involve external participants, the research team tested SecureGPT by introducing a realistic community problem and corresponding proposed solutions derived from a prior Colombian *Our Voice* project conducted with youth citizen scientists [7]. We then tested 3 different prompts designed to elicit feedback on the proposed solutions. Each prompt was tailored to simulate a particular stakeholder’s perspective. For example, we asked SecureGPT to assume the roles of a community leader, a civil engineer, and a government official reviewing the proposed solutions. We then asked SecureGPT for feedback based on these assigned roles.

### Data Collection and Analysis During Prompt Testing

#### Overview

During the preparation phase and coordinated by the second author (SAP), the research team divided into smaller working groups, each assigned a specific responsibility. The fifth (VMB) and seventh (TIS) authors led the experiments using Adobe Photoshop AI (version 25.9), while the sixth (DMC) author, along with the second (SAP) author, conducted structured testing of the large language models (SecureGPT) across different stakeholder roles and prompt configurations.

#### Testing Picture Transformation With AI

The first research group tested DALL-E’s (OpenAI) and Adobe Photoshop’s generative AI tools by using prompts to transform an image from a previous *Our Voice* project in Colombia [7]. Based on the photos and community needs, we entered image-change prompts into DALL-E and Adobe Photoshop AI. For example, for an image of a street flooded by stagnant water, we entered prompts related to removing the water or adding pavement. We aimed to test whether Adobe Photoshop AI or DALL-E could create a photo depicting the same space with an enhanced built environment. In this example, we focused on improvements that would better support adolescent health and promote walkability.

Following these iterative testing sessions, the team convened to review and compare outputs from the various AI tools. These discussions, facilitated by the first (EDVT) and second (SAP) authors, were used to synthesize key insights and refine the tools’ application in preparation for the implementation phase.

#### Testing SecureGPT

The second research group engaged in 3 rounds of iteration to explore effective prompts for eliciting feedback to strengthen community-driven solutions. The first round used 2 problems and 4 distinct community roles to assess 9 prompts, with a focus on testing a wide variety of wording. These included “Assume the role of a [role] and provide comments and concerns about this proposal” and “Provide a list of questions, concerns, and advice that a [role] would likely ask because of this situation.” The researchers gave SecureGPT 2 realistic problem scenarios based on the prior Colombian *Our Voice* project conducted with youth citizen scientists [7]. The first problem read: “The path the students walk on to get to school is filled with water. Sometimes people fall into the water while trying to cross the path. The water is very dirty and is full of diseases that can harm us. We want to create a fence to prevent people from falling in. We wish to build a bridge over the water to avoid walking over it.” They organized the responses in a roles-and-prompts matrix in Excel (Microsoft Corporation) to further discuss them as a research team.

In the second round of testing SecureGPT, the research team used the same 2 problems and gave the following prompts, which were evaluated in succession: (1) Based on the opinions of a [role], list both positive and negative feedback for this concerned community member. (2) Provide a list of questions, concerns, and advice that a [role] would ask in this situation. (3) Assume the role of [role] and provide comments and concerns about this proposal.

We tested different community roles, such as civil engineer, public health governmental official, city council leader, nonprofit leader, and school principal. In this round, the focus was to compare the 3 prompts across problem scenarios and roles. In the Excel sheet, a Notes column was used to assess each permutation of problem × prompt × role, with attention to the relevance, helpfulness, and novelty of the answers from SecureGPT.

The third round tested 1 feedback prompt: “What do you as a [role] see as 3 strengths and 3 weaknesses of this proposal? What are 5 questions you would want to ask as a follow-up?” These responses were organized in a matrix in Excel and discussed by the research team. Detailed prompt configurations and outputs from the preparation phase testing are provided in Multimedia Appendix 1.

### Implementation of an AI-Enhanced *Our Voice* Project

#### Implementation in El Pozón, Cartagena

The following section describes the implementation of the *Our Voice* project, which began in the second semester of 2024 in the El Pozón neighborhood of Cartagena, Colombia.

#### Participants and Recruitment

After receiving the study’s institutional review board (IRB) approvals (Universidad de los Andes #2024300727; referencing Stanford #40379), we asked the principal of the public school (which included grades from preschool through eleventh grade and children aged 4-19 years) to identify a group of students willing to participate in the project. He referred us to the Grupo Ecológico (Ecology Group), a group of students involved in mobilizing environmental projects. We spoke with the teacher leading that group, and she expressed interest in supporting student participation in the citizen science project. We then spoke with the students and obtained consent and assent from those willing to participate in this small first-generation study. In total, 12 students wanted to participate (7 girls and 5 boys), with a mean age of 14 (SD 1.41) years. Participants attended a public school in El Pozón, an underresourced neighborhood characterized by limited digital infrastructure. Before the study, most students had basic familiarity with smartphones for personal communication but reported no prior exposure to generative AI tools, and access to computers was limited to occasional school-based use. The participants started with the *Our Voice* discover step by walking around their community using the Discovery Tool to take photos, along with narratives of elements in their environment that diminished or facilitated their health and well-being. Then, the citizen scientists discussed their findings (discuss step) and presented them to relevant local stakeholders (advocacy step) to mobilize changes.

#### Data Collection and Analysis During Implementation

During the implementation phase, the first (EDVT) author documented structured field notes during and immediately after each facilitated session. Notes captured group discussion, decision-making, participant reflections, reactions to AI outputs, and how proposed solutions changed across iterations. After each session, notes were expanded into analytic memos to preserve context and initial interpretation and were reviewed in debriefing meetings with research subgroups drawing on expertise in public health, participatory research, and digital technologies. All sessions were audio-recorded to support accurate documentation of the process. This manuscript reports a process-focused analysis of AI integration within iterative cycles of action and reflection, using field notes, analytic memos, and archived AI prompts and outputs to examine how AI influenced solution refinement and stakeholder engagement.

To ensure transparency regarding youth agency, we summarize the mechanics of the human-AI interaction here. In brief, prompts were co-developed by the adolescent citizen scientists through facilitated consensus, and a research facilitator entered prompts and operated the AI tools during sessions while students observed. AI outputs were reviewed with the group in real time and were not used in advocacy materials without student review and group agreement. Additional procedural details and illustrative examples are reported in the “Results” section. The group discussions, prompt development, and facilitated interactions with SecureGPT and Adobe Photoshop AI were conducted in Spanish. Field notes and analytic memos were also recorded in Spanish. Prompts, AI outputs, and participant exchanges appearing in this manuscript were translated into English by the research team for publication purposes.

### Ethical Considerations

This study was reviewed and approved by the Universidad de los Andes IRB (IRB approval number 2024300727) and conducted under Stanford University’s general *Our Voice* IRB (IRB approval number 40379). Two parents or caregivers provided written informed consent for each participating adolescent, and adolescents provided assent. Participant privacy and confidentiality were protected through the deidentification of all collected data. Photographs were limited to public spaces and were reviewed before analysis and dissemination to ensure they contained no identifiable individuals or other personally identifying information. Participants were compensated with school materials (eg, water bottles, notebooks) for their time at each project stage (discovery, discussion, and advocacy), receiving approximately US $15-$20 per stage.

## Results

### Preparation Phase

The results obtained during this study phase were critical in preparing for the real-world implementation of AI during the *Our Voice* project in El Pozón.

### SecureGPT Capacity to Provide Useful Feedback

#### Prompt Testing Across Community Roles Using SecureGPT

As described above, we provided SecureGPT with prompts related to different problems and potential solutions. We then tested the different prompts in combination with different community roles.

#### Reducing Redundancy

The results of these testing processes showed that, for SecureGPT, roles with similar duties, such as city council leader and public health government official, yielded responses that did not differ significantly and thus may seem redundant. For example, when providing the first prompt to a “public health governmental official” concerning adding a fence, the SecureGPT response was, “Building a fence can help prevent people from falling into the dirty water, reducing the risk of injuries and potential exposure to harmful diseases.” Then, when providing the same prompt to a “city council leader,” a similar response was generated: “It is positive to acknowledge the safety concerns of students and community members regarding the path filled with water and to recognize the need for preventive measures, such as building a fence to prevent accidents and injuries.”

#### Framing to Elicit Engagement and Empowerment

In testing and iterating with the different prompts, the researchers found that asking SecureGPT to provide a list of questions and suggestions, rather than simple direct feedback on proposed solutions, elicited more thoughtful and reflective responses. This type of question-and-solution prompting can enhance the learning process and solution generation of citizen scientists. In addition, when SecureGPT generated questions instead of simply providing directive feedback, it could provide a launching point for further investigation, reflection, and action, which can, in turn, increase engagement and group ownership of the results. For example, when asking SecureGPT to give feedback instead of questions (prompt 1), the responses were direct pieces of advice, which we found may be less engaging and more instructional, as in the following example: “Building structures like fences and bridges may require significant financial resources that the non-profit may not currently have access to. Constructing physical barriers could potentially impact the natural ecosystem and environment surrounding the water, raising environmental concerns.” By contrast, when requesting question-oriented responses instead of simple direct feedback to improve the solutions (prompt 2), the interaction was more engaging and may produce a more positive answer-seeking group dynamic, fostering deeper understanding and engagement [20], as suggested in the following example: “What are the potential costs associated with building a fence or bridge over the water? How will the proposed fence and bridge contribute to improving the safety and well-being of the students and community? What partnerships or collaborations can be formed to support the funding and implementation of these infrastructure projects?”

#### Integrating Prompts to Elicit a More In-Depth Reasoning Process

We found that asking questions may encourage valuable insights, as has been shown in the educational literature [20]. In addition, we wanted to offer citizen scientists confirmation of the strengths of their proposals as well as identify areas for improvement. To achieve this, we tested a prompt that asked for strengths, weaknesses, and questions to enhance the proposed solution. We used the following prompt: “Assuming the role of a [role], what are three strengths and three weaknesses of this proposal? What are 5 questions you would want to ask as a follow-up?” The number of requested responses was set at this level after receiving overly long responses from SecureGPT. We selected 3 strengths, 3 weaknesses, and 5 questions to capture diversity in the responses without overwhelming future citizen scientists.

The results were promising, as SecureGPT provided a comprehensive view of the strengths and weaknesses of different solutions, along with a set of questions to guide citizen scientists in refining and enhancing their proposed solutions. For example, we obtained the result summarized in Table 1 when asking SecureGPT to assume the role of a civil engineer (see Table 1).

**Table 1:** Results of the strength, weakness, and questions prompt.

Strengths	Weaknesses	Follow-up questions
By building a bridge and fence, the proposal directly addresses the immediate safety concern of people falling into the water. Avoiding contact with the dirty water should reduce exposure to harmful diseases carried in the water, promoting overall community health. The proposed solution reflects the community’s active response in managing a pressing issue, which could inspire further self-initiated improvements.	Without a professional civil engineering plan, the bridge’s design and construction might not meet necessary safety standards, potentially leading to another danger. Building the bridge is a temporary solution that does not address the root problem of water accumulation. The proposal does not outline who will fund the construction and maintenance of these structures.	Are there any professional engineers or relevant experts involved in the design and construction of the bridge? How will the construction and ongoing maintenance of the bridge and fence be funded? Has any study been conducted to understand why the water accumulates, and has a more permanent solution been considered to address this? Have the local authorities been informed about this initiative and the larger issue at hand? Are there safety measures considered in case of potential problems with the bridge, such as a high-water level that could affect its stability?

These results informed the subsequent use of AI during the *Our Voice* discuss step. Based on the above insights, we expected that asking SecureGPT 5 specific questions, along with requesting 3 strengths and 3 weaknesses of the proposals, would add significant value to the participatory process. Additionally, given that the roles did not significantly affect the effectiveness of SecureGPT, we concluded that it was more efficient to eliminate the use of different roles and focus instead on the content of the prompts.

### DALL-E and Adobe Photoshop AI’s Capacity to Visualize Ideas

We initially tested DALL-E’s capacity to transform pictures to reflect a desired realistic future. As the seed (original) picture, we used a photo taken by an adolescent citizen scientist from a previous Colombian *Our Voice* project.

**Figure 2:**
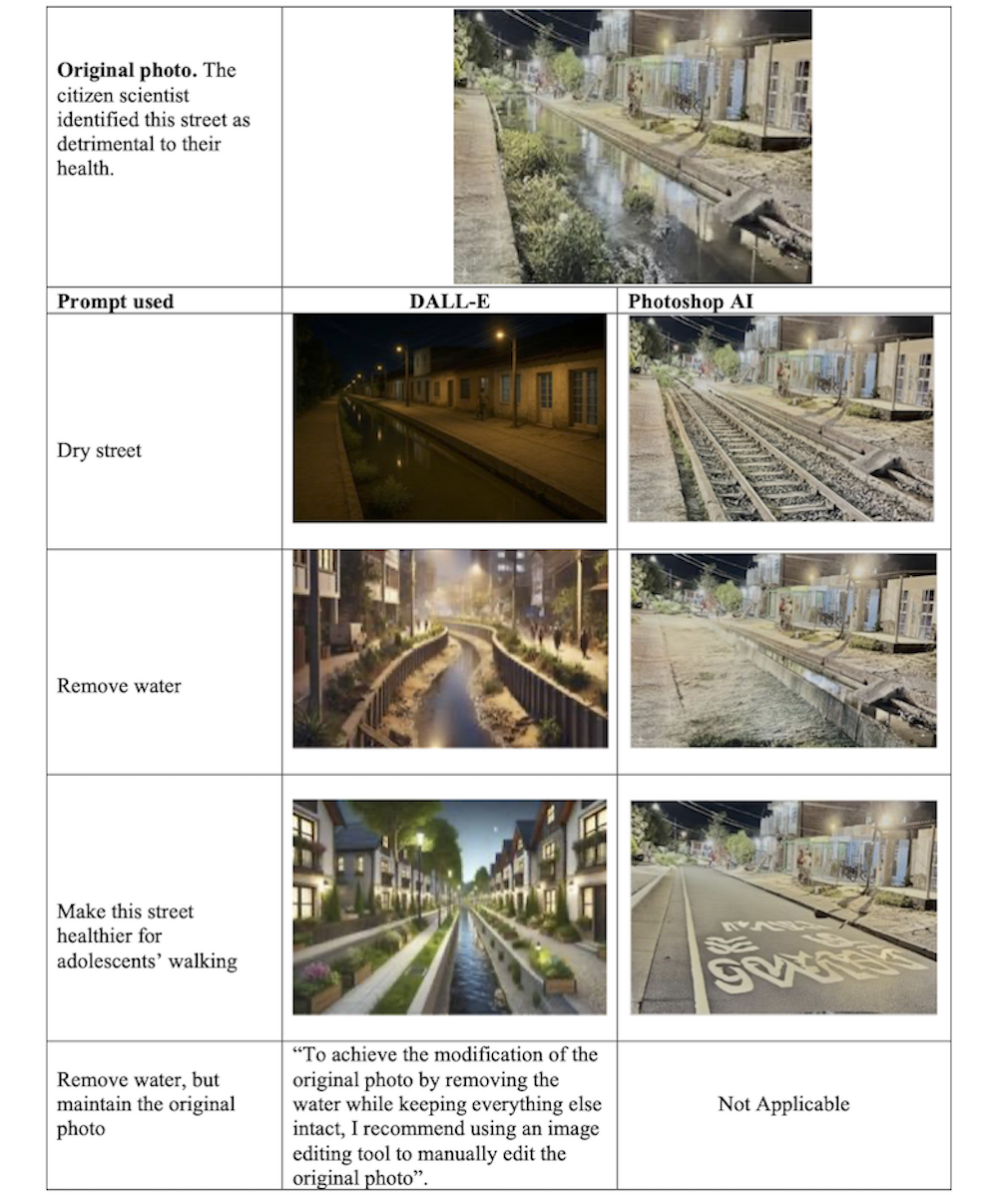
Comparison of the pictures after prompting to DALL-E and Adobe Photoshop AI.

With DALL-E, we did not find the capability to produce realistic images. Interestingly, DALL-E even suggested using an editing tool such as Adobe Photoshop. Thus, after this first iteration, we tested Adobe Photoshop AI to assess its capacity to transform photos (Figure 2).

The Adobe Photoshop AI–generated images demonstrated that picture transformations with this program were very realistic, and the process was most effective when prompts were not only specific, such as “remove water” or “dry the street,” but also more conceptual, such as “make this street healthier for adolescents to walk.” Additionally, it is important to note that a well-transformed image was often the result of an iterative process. Prompting Adobe Photoshop AI with specific requests using the lasso tool, an area-selection feature, enabled us to transform certain areas of the picture into a realistic image of the desired future. This visual representation of potential solutions can help enrich and reinforce citizen scientists’ voices, which in turn may accelerate the research-to-action process.

These findings suggest that the potential to transform pictures could significantly enhance citizen scientists’ options for discussion. We therefore determined that during the implementation phase of this project, we would use Adobe Photoshop AI and the lasso tool to select and modify specific areas of community photos collected through the Discovery Tool app.

### Utilization of AI Tools During the *Our Voice* Discuss Step

#### Overview

In this section, we report the findings from the integration of AI tools during the *Our Voice* discuss step, conducted between late 2024 and early 2025. During this phase, citizen scientists collectively analyzed their observations, prioritized key environmental health barriers, and iteratively refined proposed solutions. The primary concerns identified by the students were waste management, stagnant water, and flooding.

#### Waste Management

##### Process Overview

We offered citizen scientists access to the previously identified AI tools to help enhance their solutions for trash spilled on the school grounds.

##### Using SecureGPT to Provide Feedback to Citizen Scientist-Proposed Solutions

During the discussion meeting, we worked as a research team. Some researchers facilitated the meeting, while others used AI tools to enhance the participants’ voices. Once the citizen scientists selected a solution, a researcher, informed by the preparation phase, entered it into a SecureGPT chat dialog using a structured prompt focused on 3 strengths, 3 weaknesses, and 5 follow-up questions. The citizen scientists were particularly concerned about waste in the streets as potential sites for vector-borne infectious diseases, as well as reduced mental health in their community. The following are the results from those iterations (see Table 2).

**Table 2:** SecureGPT first iteration: feedback on the student-proposed waste management solution.

Prompts	Responses			Questions
	Strengths	Weaknesses		
The citizen scientists propose to talk with the community to promote better waste management. They propose to be a group that can speak to the community and raise awareness, with support from more influential adult actors. What do you consider are 3 strengths and weaknesses of this proposal? What are 5 questions you would ask them to reflect on and improve their proposal?	Active participation: Citizen scientists are taking an active approach to solving a significant problem in their community. Awareness: Their focus on education and awareness could contribute to a real change in the behavior of the community. Empowerment of youth: The proposal enables young people to become leaders within their community and influence adults.	Time and effort: Organizing and carrying out community meetings and awareness activities could require a lot of time and effort; can the young people maintain a long-term commitment? Waste infrastructure: Even if community attitudes change, does the community have the appropriate waste management infrastructure? Raising awareness alone may not be sufficient to change entrenched waste disposal behaviors, especially if habits are driven by structural constraints, social norms, or a lack of incentives.	How do they plan to maintain community interest in the long term? How will they ensure they obtain the support of the “more influential adult actors,” and what will they do if these actors are not willing or available to support? How will they handle the possible wear and tear on their group of citizen scientists, in terms of commitment and time? What steps will they take to ensure adequate waste management infrastructure in the community? Have they considered ways to make their initiative more sustainable, such as seeking alliances outside the community or seeking funding to support their work?	

In this iteration, the citizen scientists learned that shifts in community attitudes alone were insufficient to address the complexity of the problem after a researcher read SecureGPT’s responses verbatim.

They realized that they also needed to improve the infrastructure. Thus, after some discussion, they asked SecureGPT about waste management infrastructure, such as waste containers (see Table 3).

**Table 3:** SecureGPT’s second iteration providing structured strengths, weaknesses, and reflective questions on the proposed installation of waste containers

Prompts	Responses			Questions
	Strengths	Possible challenges/weaknesses		
And what do you think about adding the installation of containers to improve the infrastructure?	It provides a direct physical solution that makes it easier for people to handle their garbage properly. Garbage containers can reduce the problem of garbage accumulation in unwanted areas. By providing these facilities, the proposal can support improved waste management if the community shows commitment to managing and improving its garbage problem.	Containers will require regular maintenance and collection. Who will be responsible for this service? The location of the containers must be carefully considered to ensure they are accessible but also not intrusive or bothersome to the community. It requires a financial investment. Where will the funding for the containers and their maintenance come from?	Have they identified specific places suitable for the location of garbage containers? How will they make sure the containers are maintained and emptied regularly? Have they considered who will finance the garbage containers initially and who will take care of the ongoing maintenance costs? How do they plan to promote the correct use of these containers once installed? How will they handle community issues or concerns related to the location or use of the garbage containers?	

In the second iteration, the citizen scientists concluded that a combined approach of placing waste containers in appropriate locations and providing community education offered a more feasible path forward. A researcher read SecureGPT’s response aloud, and the facilitator clarified key points and corrected technical errors when they arose. The first author (EDVT), a civil engineer, also contributed relevant domain expertise. The students then wanted to use Adobe Photoshop AI to explore which types of containers they should advocate for.

##### AI-Supported Solution Visualization

We next used Adobe Photoshop AI to transform the picture. Based on the citizen scientists’ opinions, a photo taken by a citizen scientist during the walks was selected. This photo showed the waste problem in context, which is critical for picture transformation. With support from a facilitator from the research team, the citizen scientists reached consensus on the prompts they wanted to provide to Adobe Photoshop AI. During the Implementation Phase, Adobe Photoshop AI version 25.11 was used instead of version 25.9 due to a software update that occurred between the preparation phase and the implementation phase.

**Figure 3:**
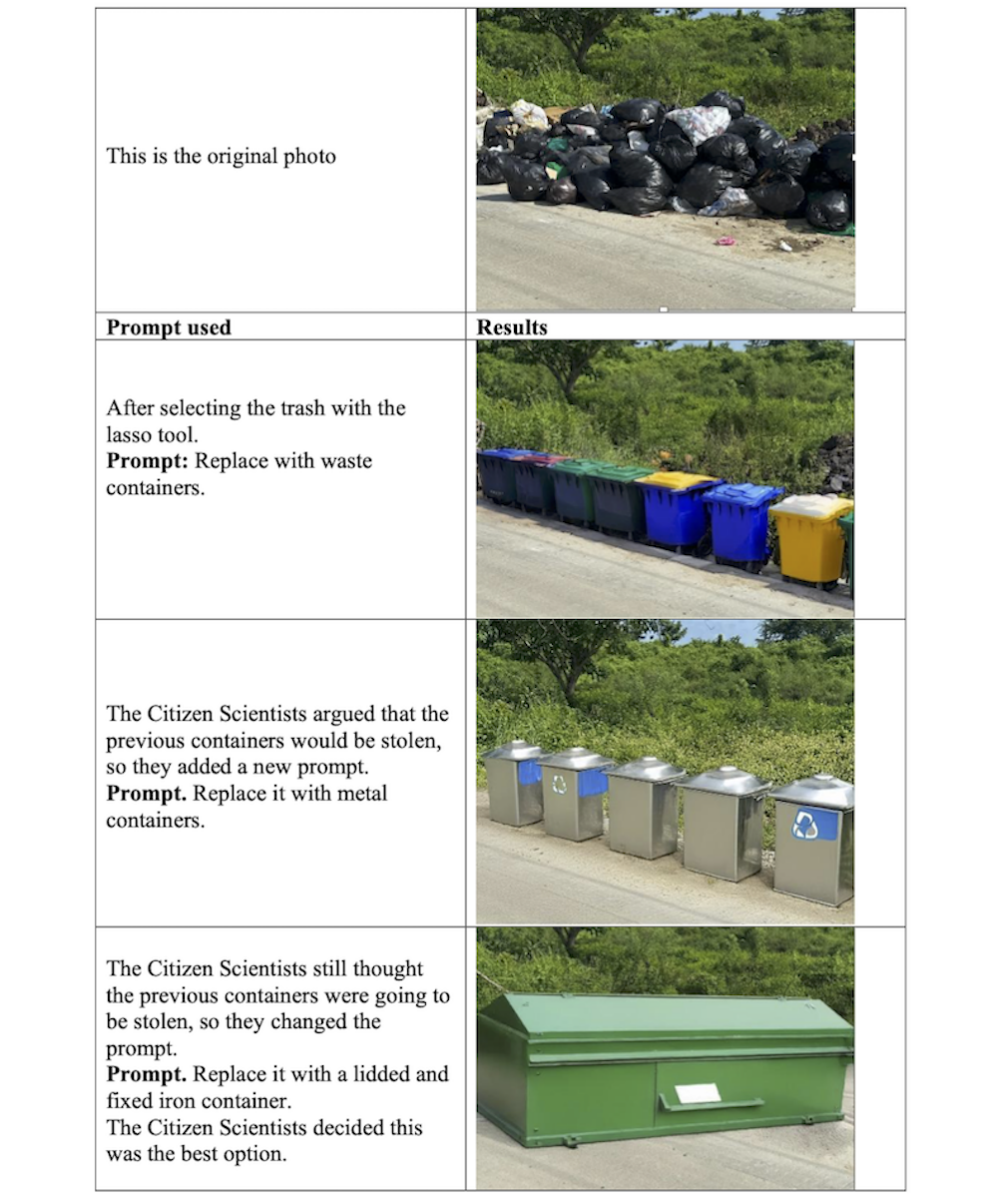
Student-guided Adobe Photoshop AI iteration visualizing waste management solutions.

The group then shared these prompts with the researcher responsible for using Adobe Photoshop AI. The researcher refined the prompts to better align with Adobe Photoshop AI and presented multiple solution options to the citizen scientists until a facilitated consensus was reached (Figure 3).

With this information, the citizen scientists had a visual representation of their desired future. They also realized that this infrastructure improvement must be accompanied by community education initiatives, such as workshops, discussions, and contests, to ensure lasting impact.

#### Stagnant Water

##### Process Overview

Given that stagnant water emerged as a second area of concern for the students, we offered citizen scientists access to specific AI tools to support and enhance their solutions to this issue, as with the previous issue.

##### Using SecureGPT to Give Feedback to Citizen Scientists–Proposed Solutions

During the project, the citizen scientists shared concerns that stagnant water increases the risk of dengue fever and other dangerous infectious diseases. From the students’ Discovery Tool data, stagnant water was frequently observed in various unfinished cement planters (or holes) in the vicinity of the school. As with the waste management issue, we introduced the citizen scientists’ proposed solution to SecureGPT using the 3-weaknesses, 3-strengths, and 5-questions prompt (Table 4).

**Table 4:** SecureGPT iteration: strengths, weaknesses, and reflective questions regarding student proposed solutions for stagnant water.

Prompts	Responses			Questions
	Strengths	Weaknesses		
For the issue of stagnant water, citizen scientists propose filling holes with materials such as sand or cement mix so that when water arrives, it does not remain and become a breeding ground for mosquitoes. This filling should be done with an analysis of whom it may affect. Efforts must be made to remove garbage from certain areas, as this contributes to water stagnation. What do you consider to be 3 strengths and weaknesses of this proposal? What are 5 questions you would ask to help them reflect and improve this proposal?	Practical action: Filling areas with material such as sand or cement mix is a practical action that can prevent the accumulation of standing water. Community effects awareness: The fact that they are looking at how the proposal may affect others in the community shows a high level of thought and consideration toward the overall impact. Broad action: The intention to eliminate trash to prevent water stagnation addresses a root cause of the problem.	Resources: Are resources such as sand and cement available? Is there work available to perform this task? Maintenance: Will they be able to keep areas filled with sand and cement over time, especially after rain and other adverse weather conditions? Long-term effects: Backfilling with certain materials could have long-term implications on soil quality and natural drainage.	What plan do you have for the long-term maintenance of the areas you have filled in? Have you considered the potential environmental implications of adding large amounts of sand or cement to the soil? How do you plan to handle any potential negative impacts on community members? How does this initiative fit into a broader plan for flood management and drainage in the community? How will you engage with the community to ensure that their needs and concerns are addressed in the filling process?	

After receiving these responses from SecureGPT (read aloud verbatim by a researcher), the citizen scientists discussed their proposed solution. Not wanting to interfere with plans for the local infrastructure (as suggested by SecureGPT), they decided against proposing to fill the holes with sand and cement, instead advocating for an easy-to-remove cement lid to cover the holes after draining them. They then used the new proposed solution to interact with Adobe Photoshop AI (version 25.11) with the mediation of a researcher.

##### AI-Supported Solution Visualization

**Figure 4:**
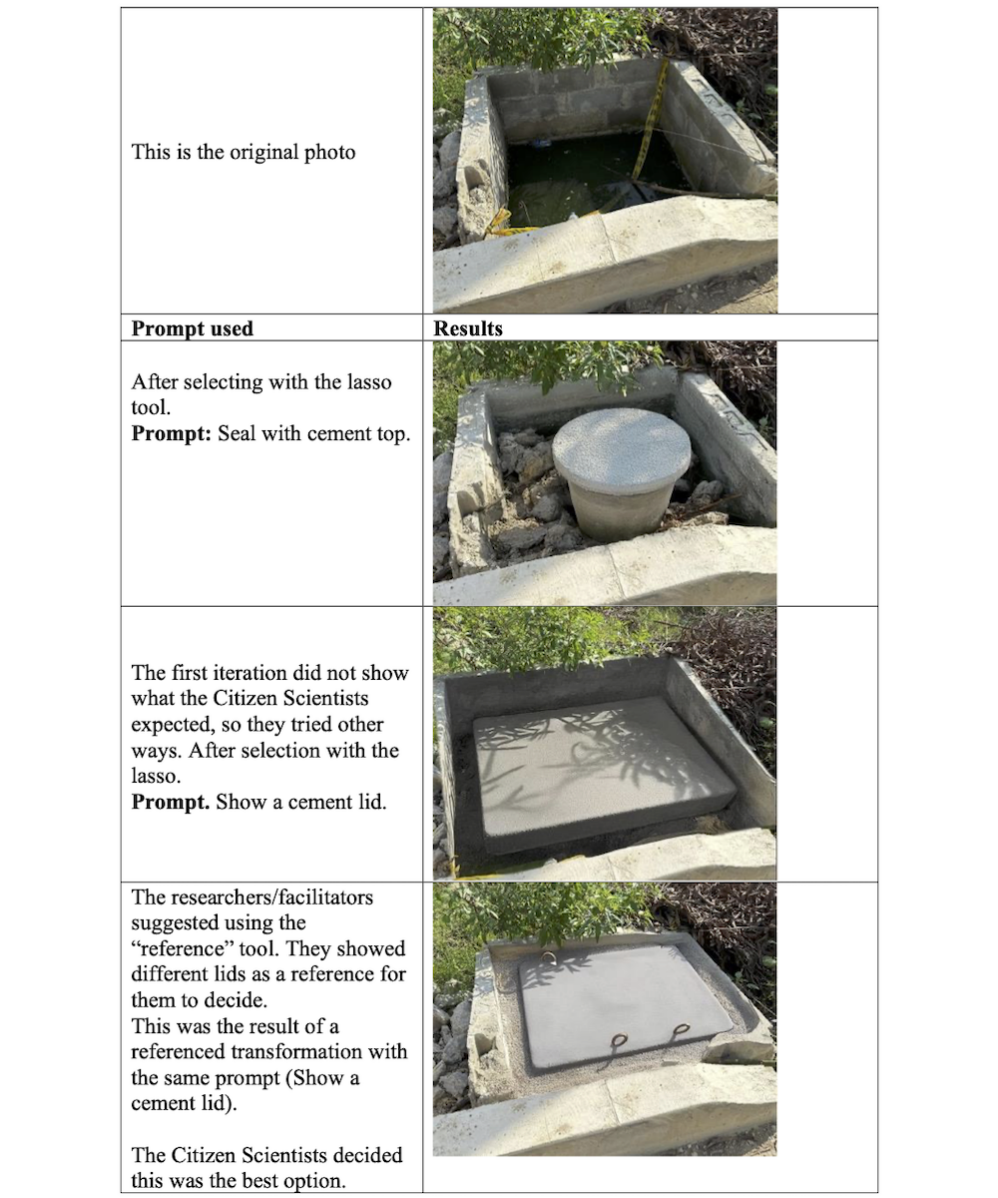
Student-guided Adobe Photoshop AI visualization of a stagnant water solution.

The first picture shown in Figure 4 shows one of the various cement planters that had remained unfinished for more than a year and represented a clear health risk. Community members had even seen dead animals in the water (see Figure 4).

After the researcher-mediated iteration with Adobe Photoshop AI, the citizen scientists proposed a solution to address the ongoing issue of planter holes without interfering with broader plans to construct a boardwalk along the entire canal. The group’s recommendation was accompanied by a tailored visual depicting the proposed solution (see Figure 4).

### The *Our Voice* Advocacy Step

Based on insights gathered from the previous *Our Voice* steps, which identified waste management and stagnant water as the most critical issues, the research team, together with the citizen scientists, invited different stakeholders and decision makers to participate in the advocacy step of the *Our Voice* method. This meeting was attended by 38 decision makers representing PaCaribe (waste management), the regional Environmental Protection Agency, the District Administrative Department of Health, Foundation Grupo Social, the Public Space Office, the Community Participation Office of the State of Bolivar (Cartagena is the capital of Bolivar), and the Nuestro Esfuerzo (Our Effort) school leadership.

During the meeting, the youth citizen scientists presented their AI-refined solutions along with AI-enhanced images illustrating them. This provided stakeholders with clear insights into the citizen scientists’ vision and recommendations. The stakeholders then organized into committees to address the pressing issues identified: waste management and stagnant water. By the end of the meeting, the stakeholders, working with the citizen scientists and project facilitators, were able to define and commit to actions for 1 and 3 months.

Among the most relevant commitments that emerged in response to the AI-enhanced proposals were (1) planning and conducting a collective walk to identify critical points for waste disposal that would inform where containers are needed; (2) placing waste containers inside the school (PaCaribe waste management group in charge) and managing them adequately (school in charge); and (3) using geo-localized coordinates of planter holes to install the lids conceptualized by the students using Adobe Photoshop AI (research team, District Administrative Department of Health, and the Police Inspector in charge).

Over the 2-month period following the advocacy step, the research team observed that the Adobe Photoshop AI–enhanced lids and the containers conceptualized by the students became the stakeholders’ objectives. As the Police Inspector noted, “We need to place those lids, those that were shown in the picture, before the rainy season starts.” In addition, PaCaribe agreed to install 2 smaller waste containers inside the school to prevent vandalism and theft. Currently, researchers, citizen scientists, and PaCaribe are in discussions to explore options for securely placing a smaller container outside the school, as it was determined that the Adobe Photoshop–created container was too large and heavy for collection and transportation. Notably, the AI-generated images continue to be referenced as aspirational goals, even if they may not represent the most feasible solutions at present. This highlights the power of visual representation to inspire stakeholders and guide discussions, as these images can serve as a motivating vision of future possibilities, even amid current practical constraints.

### AI Output Variability and Iterative Refinement

Across iterations, AI outputs varied in usefulness. Some SecureGPT responses were repetitive across stakeholder role prompts and required facilitator guidance to focus the discussion on locally relevant considerations. At times, outputs were overly long or generic, prompting refinement of the 3-3-5 prompting structure to improve specificity. Early experimentation with DALL-E for image transformation did not yield sufficiently realistic place-based modifications, leading the team to rely on Adobe Photoshop AI within a researcher-mediated workflow. These observations informed adjustments to prompting strategies and facilitation procedures throughout the implementation phase.

## Discussion

### Principal Findings

The integration of human-facilitated AI tools into an *Our Voice* Citizen Science project showed potential for supporting youth-led community engagement and advocacy efforts aimed at addressing critical environmental health issues. Recent randomized controlled trials have evaluated AI-powered chatbots as direct therapeutic agents for reducing symptoms of depression and anxiety, demonstrating short-term clinical improvements in structured individual treatment contexts. By contrast, our study examines AI not as a replacement or substitute for human therapy, but as a structured, facilitative scaffold embedded within a collective, youth-led participatory action framework [21]. This shift from AI as therapist to AI as deliberative support represents a distinct application that extends beyond symptom management toward environmental health support and civic participation. Specifically, the use of SecureGPT and Adobe Photoshop AI during the discussion and advocacy steps of the *Our Voice* method provided valuable support for refining and visually representing solutions proposed by citizen scientists, thereby facilitating clearer communication and more effective stakeholder engagement compared with prior projects in this region [6].

Notably, the images generated by Adobe Photoshop AI (version 25.11) left a lasting impression on the stakeholders, who continued to refer to the lids and containers, even though they were only visual concepts. Initial testing of these AI tools during the study’s preparation phase was essential for ensuring their agile and practical use during subsequent interactions with the citizen scientists. For instance, based on insights gained from the study preparation phase, we employed a structured prompt asking SecureGPT to identify 3 strengths, 3 weaknesses, and 5 reflective questions. This structured feedback subsequently enabled the citizen scientists to constructively refine their solutions, supporting a qualitatively expressed increase in the perceived feasibility of their proposals. Additionally, AI tools appeared to support citizen scientists in integrating information and perspectives that were typically beyond their immediate knowledge. Finally, facilitating consensus among citizen scientists regarding the prompts provided to Adobe Photoshop AI and SecureGPT was critical for improving teamwork and ensuring effective group decision-making [22-24]. Prior scholarship examining the intersection of machine learning and citizen science has primarily focused on AI as a tool for large-scale data classification and environmental monitoring. By contrast, this study explores AI as a structured cognitive and visual support tool within the deliberative and advocacy phases of participatory research. Rather than replacing human analysis, AI functioned as a structured support embedded within facilitated group processes [20,21].

In addressing both waste management and stagnant water issues, SecureGPT provided valuable feedback that aided in refining and strengthening the voices of the adolescents. For waste management, SecureGPT highlighted strengths in the adolescents’ proposed strategies, such as community awareness when accompanied by infrastructural change. It also identified important weaknesses, including over-reliance on influential adult actors, potential sustainability challenges, and the need for clearer implementation strategies. These notes prompted deeper reflection among the citizen scientists on practical considerations such as long-term sustainability and resource allocation. Similarly, for the stagnant water issue, SecureGPT’s structured feedback helped the citizen scientists critically assess their proposed interventions. For example, they initially suggested filling unfinished cement planters, but later refined the approach to installing protective lids to prevent water accumulation without spoiling public space elements. The AI-generated insights emphasized the importance of technical feasibility, long-term maintenance, and awareness of potential unintended consequences, thereby facilitating a more comprehensive understanding of the complexities involved and underscoring the health risks associated with dengue fever and other infectious diseases.

Adobe Photoshop AI was used to transform participant-generated photographs into visual representations of potential environmental changes based on the citizen scientists’ observations. These images were used during the *Our Voice* advocacy step, when the citizen scientists presented their priorities and proposed actions to stakeholders, including local authorities, health officials, and community members. In this context, the images provided a concrete reference point that stakeholders could discuss alongside the youth’s recommendations. At the same time, highly realistic AI visualizations can introduce a feasibility gap when images imply infrastructure options that are not operationally workable. If not made explicit, this gap may inflate expectations and reduce stakeholder confidence. In this study, facilitated discussion and stakeholder review were used to frame AI images as aspirational prototypes rather than technical designs and to support iterative refinement toward feasible options.

The advocacy step itself demonstrated the practical impacts of integrating AI tools into citizen science methodologies. Stakeholders, including representatives from the District Administrative Department of Health, the local police inspector, nongovernmental organizations, and waste management companies, actively engaged with the AI-refined solutions and committed to concrete short- and medium-term actions. For example, the placement of waste containers by PaCaribe and the educational workshops by the District Administrative Department of Health on dengue prevention reflect initial actions addressing the identified environmental health challenges.

### Limitations

Despite these successes, several limitations should be acknowledged. First, although Adobe Photoshop’s AI tools generated realistic visualizations, producing the final images required substantial human oversight and multiple rounds of iterative refinement. This oversight requirement aligns with broader concerns that large language models and generative systems may produce plausible yet contextually inaccurate outputs without domain-specific supervision. Ensuring that AI-generated suggestions were reviewed and contextualized by facilitators was therefore key to maintaining technical feasibility and preventing misinformation [25]. Second, Adobe Photoshop is proprietary, paid software; reliance on such platforms may limit accessibility and could exacerbate existing disparities in technological resources across users or institutions. Sustainability beyond the funded research period remains uncertain if access to proprietary AI tools is withdrawn, underscoring the need to test lower-cost or open-source alternatives. Third, the intervention was implemented within The Ecology Group, an already well-established and highly functional organization. As a result, the observed positive outcomes may not generalize to newly formed groups or teams with less cohesion, fewer resources, or less organizational structure. Fourth, documentation relied on structured field notes and analytic memos rather than verbatim transcripts, as facilitating group discussions, operating AI tools, and recording observations simultaneously create cognitive demands susceptible to recall bias. To mitigate this, field notes were expanded into analytic memos immediately after each session and reviewed in research subgroup debriefs, with audio recordings used to support documentation accuracy.

Fifth, although the first author served as group facilitator and domain expert responsible for identifying inaccuracies in AI-generated infrastructure recommendations, prompt entry and note-taking were carried out by dedicated research team members. Nevertheless, the concentration of facilitation and technical oversight within the research team introduces researcher mediation bias that may have shaped which outputs were flagged and how facilitation proceeded. Future implementations should test whether community facilitators without specialized technical training can effectively oversee AI outputs to assess replicability beyond researcher-led contexts. Sixth, the 2-month observation period following the advocacy step was initially insufficient to verify whether stakeholder commitments translated into durable environmental change. At the time of the original data collection, reported outcomes reflected documented engagement and stated intentions rather than verified infrastructural transformation. However, at the time of this resubmission, follow-up communication with school staff indicates that sustainable changes have been implemented and that students have continued to use AI tools independently to advocate for further community improvements. While encouraging, these updates are informal and were not captured through systematic follow-up, underscoring the need for future studies to incorporate structured, extended follow-up periods to rigorously assess advocacy impact. Finally, while the intervention was conducted in Spanish, AI tools can vary in accuracy and linguistic nuance across languages, which may have shaped how prompts were interpreted and outputs were generated, highlighting the need for future work to examine language-specific performance and its implications for equity.

### Future Directions

Future work should evaluate alternative tools with more intuitive interfaces, lower barriers to access, or both, and assess performance across a broader range of organizational contexts (including newly formed or less established groups) to better understand generalizability and equity considerations. In addition, future research should establish clear guidelines and best practices for effective use across diverse populations and settings, identify strategies to expand equitable access to these technologies, and develop structured facilitation processes to optimize implementation and impact. More rigorous study designs could also strengthen the evaluation of AI integration relative to the *Our Voice* approach alone. For example, matched schools or communities within the same locale could be randomly assigned to an AI-supported *Our Voice* condition, while control groups follow the traditional *Our Voice* method without AI [14].

### Conclusions

This exploratory action research study examined the integration of 2 generative AI tools within a youth-led *Our Voice* citizen science project addressing environmental health concerns in El Pozón, Cartagena, Colombia. Structured SecureGPT prompting, using a fixed strengths, weaknesses, and reflective questions (3-3-5) format, supported more systematic deliberation and feasibility-oriented refinement of adolescent-proposed solutions. Adobe Photoshop AI enabled rapid generation of place-based visual prototypes that strengthened shared understanding and provided concrete reference points during stakeholder advocacy. Across both tools, effective use depended on facilitator-mediated workflows, real-time group review, and domain-informed oversight, highlighting that these systems functioned best as structured supports within collective decision-making rather than as autonomous authorities. For participatory action and citizen science researchers, these findings suggest a practical approach for using generative AI to structure reflection, prototype solutions, and support stakeholder alignment while preserving youth agency and attending to feasibility and equity constraints.

## Data Availability

The SecureGPT prompt testing process is documented in Multimedia Appendix 1. Data generated during the implementation phase are not publicly available due to ethics committee protocols, but are available from the corresponding author (EDVT) on reasonable request. To support methodological transparency, deidentified analytic memos, the qualitative synthesis framework, structured field note templates, and selected iterative AI prompt-output sequences can also be made available on reasonable request.

## Appendix

Multimedia Appendix 1 Prompt testing matrix results.

## Abbreviations

AI: artificial Intelligence
IRB: institutional review board

## References

[1] Khan MMH, Gruebner O, Krämer A (2013). Is area affected by flood or stagnant water independently associated with poorer health outcomes in urban slums of Dhaka and adjacent rural areas?. Nat Hazards.

[2] Rosser JI, Openshaw JJ, Lin A, Taruc RR, Tela A, Tamodding N, Abdullah NPE, Amiruddin M, Buyukcangaz E, Barker SF, Turagabeci A, Leder K, Wahid I, Ansariadi, RISE Consortium (2025). Seroprevalence, incidence estimates, and environmental risk factors for dengue, chikungunya, and Zika infection amongst children living in informal urban settlements in Indonesia and Fiji. BMC Infect Dis.

[3] Khan S, Timmings C, Moore JE, Marquez C, Pyka K, Gheihman G, Straus SE (2014). The development of an online decision support tool for organizational readiness for change. Implement Sci.

[4] Kotter J (2007). Leading change: Why transformation efforts fail. Museum Management and Marketing.

[5] Yamey G (2012). What are the barriers to scaling up health interventions in low and middle income countries? A qualitative study of academic leaders in implementation science. Global Health.

[6] Montes F, Guerra AM, Higuera-Mendieta D, De La Vega-Taboada E, King AC, Banchoff A, Maturana ACR, Sarmiento OL (2022). *Our Voice* in a rural community: empowering Colombian adolescents to advocate for school community well-being through citizen science. BMC Public Health.

[7] Guerra A, De La Vega-Taboada Eduardo, Sarmiento O, Banchoff Ann, King Abby C, Stephens Dionne, Revollo Luis D, Revollo Ana P, Montes Felipe (2024). Fostering collective action for adolescent well-being: citizen science in a Colombian semi-rural area. Health Promot Int.

[8] King AC, Odunitan-Wayas FA, Chaudhury M, Rubio MA, Baiocchi M, Kolbe-Alexander T, Montes F, Banchoff A, Sarmiento OL, Bälter Katarina, Hinckson E, Chastin S, Lambert EV, González Silvia A, Guerra AM, Gelius P, Zha C, Sarabu C, Kakar PA, Fernes P, Rosas LG, Winter SJ, McClain E, Gardiner PA, On Behalf Of The *Our Voice* Global Citizen Science Research Network (2021). Community-based approaches to reducing health inequities and fostering environmental justice through global youth-engaged citizen science. Int J Environ Res Public Health.

[9] Radović S (2023). Is it only about technology? The interplay between educational technology for mathematics homework, teaching practice, and students’ activities. J Comput Educ.

[10] Mastrola M (2023). How AI can help combat climate change. The Hub.

[11] Raghav A, Singh B, Jermsittiparsert K, Raghav R, Yadav U (2024). Artificial intelligence in environmental and climate changes: a sustainable future. Maintaining a Sustainable World in the Nexus of Environmental Science and AI.

[12] Rowe JP, Lester JC (2020). Artificial intelligence for personalized preventive adolescent healthcare. J Adolesc Health.

[13] Posner T, Fei-Fei L (2020). AI will change the world, so it’s time to change AI. Nature.

[14] Opel N, Breakspear M (2026). Transforming mental health research and care through artificial intelligence. Science.

[15] King AC, Doueiri ZN, Kaulberg A, Goldman Rosas L (2025). The promise and perils of artificial intelligence in advancing participatory science and health equity in public health. JMIR Public Health Surveill.

[16] Haenlein M, Kaplan A (2019). A brief history of artificial intelligence: on the past, present, and future of artificial intelligence. California Management Review.

[17] Yuwono B (2023). The transformation of digital imaging in Photoshop with the presence of artificial intelligence generators.

[18] Ayala-García J, Meisel-Roca A (2017). Cartagena Libre de Pobreza Extrema en el 2033. Banco de la Republica.

[19] Bradbury H, Waddell S, O’ Brien K, Apgar M, Teehankee B, Fazey I (2019). A call to action research for transformations: the times demand it. Action Research.

[20] Lotfian M, Ingensand J, Brovelli MA (2021). The partnership of citizen science and machine learning: benefits, risks, and future challenges for engagement, data collection, and data quality. Sustainability.

[21] Heinz MV, Mackin DM, Trudeau BM, Bhattacharya S, Wang Y, Banta HA, Jewett AD, Salzhauer AJ, Griffin TZ, Jacobson NC (2025). Randomized trial of a generative AI chatbot for mental health treatment. NEJM AI.

[22] Neenan M (2008). Using Socratic questioning in coaching. J Rat-Emo Cognitive-Behav Ther.

[23] Marvin F (2002). Consensus is primary to group facilitation. Group Facilitation.

[24] Pérez I, Cabrerizo F, Alonso S, Dong Y, Chiclana F, Herrera-Viedma E (2018). On dynamic consensus processes in group decision making problems. Information Sciences.

[25] Siddals S, Torous J, Coxon A (2024). "It happened to be the perfect thing": experiences of generative AI chatbots for mental health. Npj Ment Health Res.

